# The Role of Cytotoxic T-Lymphocyte Antigen 4 in the Pathogenesis of Multiple Sclerosis

**DOI:** 10.3390/genes13081319

**Published:** 2022-07-24

**Authors:** Maria Sofia Basile, Placido Bramanti, Emanuela Mazzon

**Affiliations:** IRCCS Centro Neurolesi “Bonino-Pulejo”, Via Provinciale Palermo, Contrada Casazza, 98124 Messina, Italy; placido.bramanti@irccsme.it (P.B.); emanuela.mazzon@irccsme.it (E.M.)

**Keywords:** multiple sclerosis, CTLA-4, immune checkpoint, autoimmunity, peripheral blood mononuclear cells, T-cells maturation, EAE animal models, genetic predisposition, genetic polymorphisms, abatacept

## Abstract

Multiple sclerosis (MS) is an autoimmune neurodegenerative disorder of the central nervous system that presents heterogeneous clinical manifestations and course. It has been shown that different immune checkpoints, including Cytotoxic T-Lymphocyte Antigen 4 (CTLA-4), can be involved in the pathogenesis of MS. CTLA-4 is a critical regulator of T-cell homeostasis and self-tolerance and represents a key inhibitor of autoimmunity. In this scopingreview, we resume the current preclinical and clinical studies investigating the role of CTLA-4 in MS with different approaches. While some of these studies assessed the expression levels of CTLA-4 on T cells by comparing MS patients with healthy controls, others focused on the evaluation of the effects of common MS therapies on CTLA-4 modulation or on the study of the CTLA-4 blockade or deficiency in experimental autoimmune encephalomyelitis models. Moreover, other studies in this field aimed to discover if the *CTLA-4* gene might be involved in the predisposition to MS, whereas others evaluated the effects of treatment with CTLA4-Ig in MS. Although these results are of great interest, they are often conflicting. Therefore, further studies are needed to reveal the exact mechanisms underlying the action of a crucial immune checkpoint such as CTLA-4 in MS to identify novel immunotherapeutic strategies for MS patients.

## 1. Introduction

Multiple sclerosis (MS) is an autoimmune neurodegenerative disorder of the central nervous system (CNS) characterized by inflammatory demyelination and axonal transection, which usually occurs in young adults, with a mean age of onset of 20–30 years, and can cause physical disability, cognitive impairment, and a reduced quality of life [1].

Based on data from 2020, the prevalence of MS worldwide is 35.9 per 100,000 people, and females are twice as likely to present MS as males [2]. Generally, the life expectancy of those with MS is lower than the general population (75.9 years in an MS population in comparison to 83.4 years in a matched population) [1].

MS shows heterogeneous clinical manifestations and course [3]. According to the National Multiple Sclerosis Society Advisory Committee on Clinical Trials in MS, there are four clinical courses of MS: relapsing–remitting MS (RRMS), secondary progressive MS (SPMS), primary progressive MS (PPMS), and progressive relapsing MS (PRMS) [3]. RRMS occurs in approximately 85% of patients and is characterized by the development of relapses at irregular intervals with a total or partial neurological recovery [3]. SPMS is characterized by progressive, irreversible disability independent from the occurrence of relapses and ~2–3% of RRMS patients convert to SPMS each year [3]. PPMS is characterized by disease progression from the onset, leading to gradual, progressive, and permanent neurological deficits for more than 1 year without relapses and is present in nearly 10–15% of patients [3]. PRMS is infrequent and is characterized by progressive disease, with acute relapses (with or without complete clinical recovery) and periods of continued progression amongst relapses [3]. Furthermore, a revision of these phenotypes has been introduced to include clinically isolated syndrome (CIS) and thus indicate the patients whose early clinical presentation is characterized by inflammatory demyelination that might be MS without fully accomplishing its diagnostic criteria [3]. In addition, each subtype of MS might be classified as active or not active on the basis of the development of relapses or lesions detected by magnetic resonance imaging (MRI) [3]. MS can affect many functions involving cognitive, emotional, motoric, sensory, or visual domains [4]. Of note, cognitive impairment, fatigue, depression, anxiety, and pain can often co-occur in MS [5,6]. 

In particular, cognitive impairment affects 40–65% of MS patients, substantially contributing to their disability status [7]. It is known that cognitive impairment is more common and severe in the progressive forms of MS; however, it may appear in the early clinical stages of MS, generally manifesting in deficits of long-term memory, attention and concentration, executive functioning, efficiency of information processing, and processing speed [7]. The most commonly affected cognitive domains are cognitive processing speed and episodic memory [8]. An interesting facet that should be considered is that different psychopathological conditions are characterized by severe impulsivity problems that can contribute to causing disability due to poor regulation and control, which can become worse in the presence of emotional cues [9]. Interestingly, emotions could ameliorate or disturb motor inhibition ability [9]. Of note, a great deal of evidence, additionally supported by studies of brain-damaged patients, has suggested that the superior temporal sulcus could have a role in attentional orienting, whereas the amygdala could be involved in jointly elaborating gaze and emotional expressions [10]. 

A disruption of the blood–brain barrier (BBB), multifocal inflammation, demyelination, oligodendrocyte loss, reactive gliosis, and axonal degeneration are involved in the MS pathological process [11]. 

Of note, inflammatory demyelination and neurodegeneration have been associated with neuropsychological deficits in MS patients [7]. In addition, neuroanatomical changes, pro-inflammatory cytokines, the dysregulation of monoaminergic pathways, and a hyperactive hypothalamic–pituitary–adrenal axis have been associated with the co-occurrence of fatigue, cognitive impairment, depression, and pain in MS [5].

It should be noted that aberrant fear conditioning is largely considered a mechanism involved in different psychiatric diseases, including depression [12]. Interestingly, it has been shown that human fear conditioning can be regulated by a complex interplay between the CNS and the autonomic nervous system [12]. 

Of note, cascading high-level cognitive structures, in particular the prefrontal cortex, can influence the activities of the amygdala and hippocampus, leading to neurovisceral fear responses via sympathetic and parasympathetic projections that regulate heart-related dynamics [12,13].

In addition, MRI studies have demonstrated that extensive alterations to brain networks can contribute to cognitive dysfunction, and that grey matter atrophy represents an early sign of possible future cognitive decline [8]. In particular, the burden of cortical lesions and tissue loss could be considered among the most important structural alterations associated with cognitive impairment in RRMS [7]. Moreover, symptoms of depression have been associated with alterations in frontostriatal monoamines implicated in reinforcement learning [14]. 

Although MS’s etiology is not clear, it has been hypothesized that MS could be associated with different genetic factors, such as the major histocompatibility complex *HLA-DRB1* locus, and with different lifestyle and environmental factors, including tobacco smoking, childhood obesity, low vitamin D serum levels, UV radiation, and Epstein-Barr virus infection [1,15].

In particular, low levels of Vitamin D have been found in neonatal and adult cohorts of MS patients [16]. Moreover, the serum Vitamin D biomarker can be considered an important risk factor for long-term MS activity and progression in the early course of the disease, and it can predict new active lesions and the relapse rate [16]. However, vitamin D supplementation seems to have no therapeutic effect on the EDSS score in MS patients [17,18]. Instead, recent preclinical evidence has suggested that a combined therapy consisting of the use of Vitamin D3 and tolerogenic dendritic cells (tolDC) associated with interferon (IFN) β could represent a promising strategy for treating MS [19].

Of note, it has been shown that alterations of reduction-oxidation homeostasis frequently occurs in MS patients [6].

Furthermore, the presence of kynurenine system activation has been shown in different neurodegenerative diseases; particularly, alterations of the kynurenine pathway metabolites have been found in MS [20,21]. Indeed, it has been shown that neurotoxic kynurenines were augmented in MS, whereas there were mixed results regarding the neuromodulatory kynurenines in MS [22]. 

Moreover, it has been found that pro-inflammatory cytokines, such as interleukin (IL)-1, IL-12, IL-17, IL-22, tumor necrosis factor-α, and IFN-γ are elevated in MS and could contribute to the demyelination of the neural pathways [22,23]. Conversely, anti-inflammatory cytokines, including IL-4 and IL-10, are decreased in MS [22,23]. Overall, it has been suggested that a closely regulated endogenous network consisting of proinflammatory and anti-inflammatory cytokines and other cellular and soluble mediators could regulate the onset and the progression of the disease and could also beinvolved in its response to treatment, thus suggesting novel possible candidates for diagnostic markers and therapeutic targets [24,25]. 

Furthermore, it has been suggested that the mammalian target of the rapamycin (mTOR) network could be significantly involved in the etiopathogenesis of MS [26,27].

Currently, the diagnostic criteria for MS rely on the detection of lesions in the CNS that show a dissemination in space and time [28]. Moreover, alternative diagnoses that could clinically or radiologically mimic MS must be excluded [28].

Nowadays, MS treatment is multidisciplinary and comprises disease-modifying therapies (DMTs), the treatment of acute relapses, the management of comorbidities, symptomatic treatments, rehabilitative interventions, psychological assistance, and lifestyle modifications [1]. Until July 2020, nine classes of DMTs were approved for the treatment of MS: interferons, glatiramer acetate, teriflunomide, sphingosine 1-phosphate [S1P] receptor modulators, fumarates, cladribine, natalizumab, ocrelizumab, and alemtuzumab [1]. DMT treatment can decrease the annualized relapse rate by 29% to 68% in comparison to placebos or active comparators [1]. 

At the beginning of the COVID-19 pandemic, there were many reserves concerning the treatment of MS patients with disease-modifying drugs owing to the risk of severe COVID-19, and many neurologists were hesitant to start therapy or change it because of the fear of COVID-19 infection [29]. Nevertheless, delaying treatment or switching one type of drug with another one with a stronger efficacy could cause MS undertreatment and the accrual of disability [29]. Therefore, an individual evaluation of the risk of a severe COVID-19 infection should be performed for MS patients by defining the most appropriate vaccination schedule, considering the treatment with the disease-modifying drugs, and comparing the possible risks in avoiding this treatment with its benefits [29]. 

Interestingly, a promising therapeutic strategy for MS could be represented by statins, which are first-choice agents for the primary and secondary prevention of cardiovascular diseases and can exert anti-inflammatory and antioxidant action [30,31]. Trials investigating the effects of statins on SPMS have shown an encouraging effect on the progression of disability, supporting their potential immune-modulatory and neuroprotective role [31].

It is unknown whether part of the effects of statins could be associated with their brain penetration or are mediated by a cytokine decrease in the periphery [30].

Moreover, among the novel therapies that are currently being evaluated there are cell-based therapies, which involve hematopoietic and mesenchymal stem cells, and remyelination therapies with the potential to contribute to ameliorating MS treatments [1]. Different studies with various compounds, including biotin, clemastine, opicinumab, and mesenchymal stem cells, have evaluated the potential of remyelination; however, there are limited data regarding the effectiveness of this treatment [1]. Furthermore, although high-dose immunosuppressive therapy with autologous hematopoietic stem cell transplantation is not included in the routine practice, there is evidence that it could exert therapeutic action [1]. 

MS is particularly suitable to personalized treatment due to its large range of clinical presentations and therapeutic responses [32]. The approach to personalized MS therapy relies on evidence-based prognostication, an initial treatment decision, and the assessment of early treatment responses to establish the need to change therapies [32]. Prognostication is the basis of personalized treatment and allows for the grouping of subjects according to their demographic and environmental characteristics, clinical features, MRI measures, and biomarkers [32]. 

A major problem in the management of MS is that despite the availability of the previously discussed disease-modifying treatments, many MS patients experience a persistent progression of disease, clinical relapses, disease activity, and adverse effects [33]. Gene expression, proteomic, or genomic approaches can be used to find novel biomarkers with a predictive value towards identifying a beneficial or poor clinical response to therapy and treatment risks [33]. Of note, the variety of potential promising molecular markers is quickly growing [33].

According to this line of research, it has been suggested that polymorphisms in certain genes (*CD46*, *CD58*, *FHIT*, *IRF5*, *GAPVD1*, *GPC5*, *GRBRB3*, *MxA, PELI3*, and *ZNF697*) could be potential predictive markers of a response to IFN-β treatment in RRMS patients [34]. These data are of great interest since many patients show suboptimal responses to this treatment and approximately 20–50% of them are non-responders, even though it is one of the first-line treatments for MS patients [34].

In addition, glatiramer acetate, in spite of the fact that it represents an important first-line treatment for MS patients, shows a high variability in responses among patients, with a response rate of nearly 30–55% [35]. Of note, it has been shown that genetic factors, including polymorphisms in the genes implicated in MS pathogenesis, could influence this variability in the drugs’ effectiveness [35]. In particular, it has been suggested that there is a relationship between the effectiveness of glatiramer acetate treatment and the presence of polymorphisms in these genes: *CD86*, *CLEC16A*, *CTSS*, *EOMES*, *MBP*, *FAS*, *TRBC1*, *IL1R1*, *IL12RB2*, *IL22RA2*, *PTPRT*, *PVT1*, *ALOX5AP*, *MAGI2*, *ZAK*, *RFPL3*, *UVRAG*, *SLC1A4*, and HLA-DRB1*1501 [35]. Therefore, the identification of polymorphisms in the above-mentioned genes might be utilized as a predictive marker of glatiramer acetate response in MS patients [35].

Moreover, a percentage of patients do not respond to other first-line treatments (dimethyl fumarate and teriflunomide) and to several second-line treatments (natalizumab, fingolimod, alemtuzumab, cladribine, siponimod, and ocrelizumab), although these drugs have a high rate of response and can decrease the annual attack rate by 31–69% and the progression of the disease by up to 66% [36]. 

Noteworthily, it has been shown that polymorphisms in the *GSTP1*, *ITGA4*, *NQO1*, *AKT1*, and *GP6* genes for treatment with natalizumab; *ZMIZ1* for fingolimod and dimethyl fumarate; *ADA*, for cladribine; and *NOX3*, for dimethyl fumarate, could be predictive markers of treatment responsiveness for patients with MS [36]. In addition, it has been shown that a specific gene expression profile of CD4+ T cells could characterize the pharmacological responsiveness to natalizumab in MS patients [37]. 

Molecular biomarkers are very important for personalized therapy and should be characterized by high-sensitivity and specificity and by an easy, cost-effective, reproducible, and non-invasive detection method [38]. 

To date, the use of biomarkers has helped MS diagnosis and prognosis and the monitoring of treatment responsiveness as well as the assessment of the risk of side effects [38]. Hence, the identification of novel biomarkers and novel tailored therapeutic targets for MS, along with the characterization of the pathogenetic pathways involved, are of the utmost importance [38,39].

Among the biomarkers currently considered, there are oligoclonal bands and the IgG index, anti-aquaporin-4-antibodies, and neutralizing antibodies against IFN-β and natalizumab, but also anti-John Cunningham virus and anti-varicella-zoster virus antibodies [38]. Moreover, neurofilament light and chitinase-3-like-1 may be promising potential biomarkers [40,41].

In addition, different oxidative enzymes, antioxidative enzymes, and redox degradation products have been suggested to be potential promising biomarkers for MS diagnosis [42]. 

Considering that the identification of novel biomarkers and targets and personalized medicine are fundamental priorities for MS and since MS is an autoimmune disorder, the recognition of receptors and ligands that decrease the T cells activity can be promising for MS targeted therapy; therefore, the evaluation of the role of Cytotoxic T-lymphocyte-associated protein 4 (CTLA-4) in MS deserves particular attention [11,43]. 

CTLA-4 is an inhibitory receptor mainly expressed by T-cells that belongs to the CD28 immunoglobulin subfamily and binds to CD80 and CD86, also known as B7-1 and B7-2, which are usually present on the surface of antigen-presenting cells (APCs) [44]. CD80 and CD86 can bind to CD28 or CTLA-4, respectively, thus generating a co-stimulatory or a co-inhibitory response [44]. Due to its dampening effect, CTLA-4 is a critical regulator of T-cell homeostasis and self-tolerance and represents a key inhibitor of autoimmunity [44,45]. 

CTLA-4 mediates its immunomodulatory effects by binding to CD80 and CD86 because it competes with CD28 [46]. This binding increases the threshold activation of T cells, so the immune response is significantly reduced [46]. Some evidence highlights the possibility that CTLA-4 prevents the maturation of T-cells inhibiting the activation of Akt pathway induced by CD3. It is important to mention that CTLA-4 can influence the Akt pathway but seems to preserve the activity of PI3K/ [47].

The therapeutic targeting of immune checkpoints has attracted considerable attention in cancer immunotherapy, particularly concerning CTLA-4 and programmed cell death 1 (PD-1) [48]. Noteworthily, in autoimmunity, these pathways might be targeted to the contrary effect to suppress the excessive immune response [48]. 

Indeed, manipulating the signals between APCs and T-cells could represent a potential clinically relevant strategy [49]. In certain conditions, the administration of decoy coinhibitory receptors, such as CTLA-4 Ig or mAb, against coinhibitory molecules could inhibit the responses of self-reactive T cells in autoimmune disorders, thus suggesting that modulating the coinhibitory signals could be a promising approach to induce tolerance in autoimmune diseases [49].

On the other hand, immune checkpoint therapy, which aims to target regulatory pathways in T cells to increase antitumor immune responses, has led to a significant clinical progression, representing a novel tool for fighting cancer [50]. Starting with the approval of anti-CTLA-4 antibodies for advanced-stage melanoma in 2011, immune checkpoint inhibitors, which now also comprise antibodies against PD-1 and its ligand (PD-L1), have been rapidly approved by the US Food and Drug Administration (FDA) for the treatment of different cancers [51]. Ipilimumab, a human CTLA-4-blocking antibody, is the only FDA-approved CTLA-4 inhibitor [52].

An interesting piece of data that has contributed to paving the way to the study of the role of CTLA-4 and of other immune checkpoints in the pathogenesis of MS concerns the fact that several immune checkpoint inhibitors, including CTLA-4 inhibitors, could trigger or exacerbate certain immune mediated diseases, including MS [53,54,55,56,57,58,59]. Indeed, it seems that immune checkpoint inhibitors might induce epitope spreading and a higher T cell response, which could act as a trigger for some immune-mediated diseases [53]. Interestingly, Garcia and colleagues have investigated the outcomes of documented cases of MS relapse following immune checkpoint inhibitor-treatment and they have found that ipilimumab could be associated with reported cases of MS [53]. 

Of note, during the last few years it has been shown that different immune checkpoints can be involved in the pathogenesis of autoimmune diseases and neurodegenerative diseases, including MS, and could represent novel biomarkers, targets, or candidate genes for disease susceptibility [45,60,61,62,63,64,65,66,67,68,69,70]. 

Indeed, it is known that the immune system is well-orchestrated and that its balance can be regulated by immune checkpoints, which can act as co-stimulatory and co-inhibitory molecules and are fundamental for the maintenance of self-tolerance [45,71]. Thus, either an exaggerated co-stimulation or an insufficient co-inhibition might result in the development of autoimmune diseases, including MS [45,72]. 

It is worth mentioning that the immune regulatory function of co-inhibitory receptors such as CTLA-4, PD-1, LAG-3, TIM-3, and TIGIT was initially identified in autoimmune disorder models, since their blockade or deficiency led to the induction or exacerbation of the disease [73].

In addition, it has been suggested that CTLA-4, PD-1, TIGIT, and TIM-3 can inhibit both autoreactive T cells and the development of CNS autoimmunity [74]. 

Furthermore, it has been shown that PD-1/PD-L1 exert an immunoregulatory action in different immune cells, such as T cells, B cells, natural killer (NK) cells, dendritic cells (DCs), and macrophages/microglia in MS and experimental autoimmune encephalomyelitis (EAE) models [75]. Moreover, PD-1/PD-L1 can negatively regulate immune responses and is implicated in the therapeutic efficacy of disease-modifying therapies for MS [75]. 

Interestingly, numerous studies have focused on the evaluation of the role of CTLA-4 in MS. 

**Figure 1:**
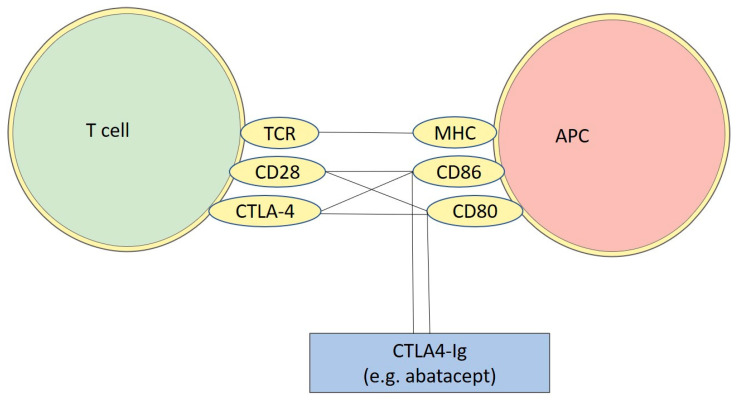
Schematic figure representing the binding sites of CTLA4-Ig. As illustrated, Cytotoxic T-Lymphocyte Antigen 4 (CTLA-4) share with CD28, located on T-cells, the possibility of binding with CD86 and CD80, located on APC (antigen-presenting cell). Abatacept can be used as a possible treatment for multiple sclerosis (MS), since it binds to CD86 and CD80 in a similar way as CD28 and CTLA-4. This leads to a competition for the binding site, resulting in a blockage of T-cell maturation where the T-cell are unable to interact with APCTCR: T cell receptor; MHC: major histocompatibility complex.

Of note, it has been shown that the *CTLA-4* gene might be involved in the predisposition to MS and that a defect in CTLA-4 signaling could be implicated in the immune dysregulation that occurs in MS patients [76]. Moreover, the potential use of abatacept—a fusion protein consisting of the extracellular domain of CTLA-4 and the Fc portion of Ig-G (CTLA4-Ig)—on MS patients has been evaluated [77,78,79]. In particular, abatacept can bind to the costimulatory ligands CD80 and CD86 and block their interaction with the CD28 and CTLA-4 receptors expressed by T cells; thus, it leads to the inhibition of T cell activation and function, and it is effective in the treatment of certain autoimmune diseases but not in others [79] (Figure 1).

Identifying the role of CTLA-4 in the pathogenesis of MS and discovering the potential value of drugs targeting this crucial immune checkpoint might shed light on new perspectives in the management of MS. Therefore, herein, we review the current preclinical and clinical studies investigating the role of CTLA-4 in MS.

## 2. Preclinical Studies

### 2.1. In Silico, In Vitro, and Ex Vivo Studies

Different preclinical in silico, in vitro, and ex vivo studies have investigated the role of CTLA-4 in MS. The most important results from these studies have been illustrated in Table 1.

Mena and Rohovsky-Kochan have examined the expression of CTLA-4 on peripheral blood T and B lymphocytes and monocytes from patients with MS and healthy controls [80]. They found similar expressions of CTLA-4 on CD4+ and CD8+ T cells between the treated and untreated patients with MS [80]. Furthermore, there were no significant differences in the expression of CTLA-4 onmonocytes or CD4+ and CD8+ T cells between the treated or untreated MS patients compared with healthy controls [80]. In addition, analyzing the peripheral blood mononuclear cells (PBMC) of an MS patient with a very rapidly progressing disease, the authors found that the expression of CTLA-4 was raised on both T-cell subsets at all the times evaluated [80]. 

According to this line of research, Lavon and colleagues have analyzed the expression levels of some co-inhibitory receptors, including CTLA-4, in PBMC from healthy controls and patients with untreated MS and they found that the CTLA-4 levels were not statistically significantly different when comparing the two groups [81].

Oliveira et al. have investigated the role of CTLA-4 engagement in myelin basic protein (MBP) responses in MS patients compared to healthy individuals [76]. They did not find any significant difference in the CTLA-4 expression levels on T cells in the PBMC isolated ex vivo from MS patients in comparison to healthy subjects [76]. In addition, they demonstrated that the blockade of CTLA-4-mediated signaling during the stimulation of MBP-reactive T cells from healthy subjects increased proliferative and cytokine responses, whereas blocking CTLA-4 in MS patients had fewer effects [76]. These data support the hypothesis that a reduced sensitivity to the negative and regulatory CTLA-4-mediated signaling could characterize MS patients, distinguishing them from healthy controls, and in particular, the failure of the anergy of hMBP-reactive T cells, which might be associated with a defect in CTLA-4-mediated signaling, could occur in these patients [76]. 

Alternatively, Mohammadzadeh et al. have explored the expression pattern of certain inhibitory receptors, including CTLA-4, analyzing the PBMC of RRMS patients in comparison with those of healthy controls, and they showed that the expression of CTLA-4 was decreased in RRMS patients compared with the controls [82]. Hence, this study supported the hypothesis that the downregulation of inhibitory receptors might be involved in the dysregulation of immune tolerance against CNS autoantigens; therefore, these inhibitory receptors could represent promising gene targets for MS [82].

In addition, Wang et al. have evaluated the plasma concentrations of the soluble form of CTLA-4 (sCTLA-4) in MS patients, neuromyelitis optica (NMO) patients, and controls, and they found that the levels of sCTLA-4 were decreased in MS patients in comparison to the controls and that sCTLA-4 did not correlate with the EDSS score in MS and NMO patients [83].

Moreover, Eschborn et al. have analyzed the immune signatures in peripheral blood and CSF, comparing RRMS patients, PPMS patients, and controls to explore age-related immunologic alterations in MS [84]. They found that while healthy donors showed a strong age-dependent decrease in the expression of different immunoregulatory molecules, including CTLA-4, on memory CD8 T cells, this age-dependent regulation was abrogated in MS patients [84]. Indeed, young patients already showed an expression level of these molecules similar to that from old MS patients or to that from old healthy donors [84].

Kosmaczewska et al. have assessed the membrane/surface (m) and cytoplasmic (c) expressions of CTLA-4 in freshly isolated peripheral blood CD4+ T lymphocytes from RRMS and SPMS patients in clinical remission compared with healthy controls, and they have also evaluated the ability of these cells to express these molecules subsequently to ex vivo stimulation with anti-CD3+ rIL-2 [85]. Conversely to the previous studies, they have shown a significantly increased median percentage of freshly isolated peripheral blood CD4+/CTLA-4+ T cells from both groups of MS patients, which was more marked in RRMS patients [85]. Instead, a negligible proportion of these cells was observed in the controls [85]. Moreover, the CD4+ T cells from both groups of MS patients showed a comparable inability to achieve normal levels of surface CTLA-4 expression when re-stimulated [85]. Indeed, no induction of surface CTLA-4 expression over pre-stimulation levels was observed in SPMS patients, and it even declined in RRMS patients [85].

Derakhshani et al. have used single-cell RNA-seq data to evaluate the *CTLA-4* gene expression in different PBMC cell types of patients with MS and they showed that *CTLA-4* has a substantial expression in the naïve T cells, Tregs, and activated CD8^+^ T cells [86]. Moreover, they have evaluated CTLA-4 expression in PBMC samples comparing healthy controls with naïve patients (RRMS patients who did not receive any treatment) and they found that CTLA-4 expression in naïve patients is more reduced than in healthy subjects [86]. Furthermore, Derakhshani et al. have assessed the CTLA-4 expression in PBMC samples from RRMS patients who were treated with different drugs, such as fingolimod, IFNβ-1α, glatiramer acetate, and dimethyl fumarate, in comparison with samples of naïve patients and healthy controls [86]. Interestingly, they found that several treatments, in particular fingolimod, can induce the expression of *CTLA-4* and that its higher expression or function could contribute to reducing the responses of autoreactive T cells and to inhibiting autoimmune diseases, such as MS [86]. 

Hallal-longo et al. have explored the T cell response to myelin antigens and a nonspecific mitogen as well as the expression of CTLA-4 and Fas molecules in the PBMC of MS patients, either treated or untreated with IFN-β [87]. They have shown that IFN-β reduced the proliferative response to MBP and myelin and augmented the expression of the CTLA-4 intracellular molecules, thus suggesting that the rise in the CTLA-4 molecules in MS patients could lead to lymphocyte apoptosis and thus shed light on the potential mechanisms involved in the therapeutic response to IFN-β [87].

Sellebjerg and colleagues have discovered that the percentage of CD4^+^CD25^high^ T cells expressing CTLA-4 was significantly different when comparing untreated MS patients with healthy controls [88]. While MS patients showed an increased percentage of CD4^+^CD25^high^ T cells with a total (intracellular + surface) expression of CTLA-4 in comparison to controls, independently of IFN-β treatment, the percentage of CD25^high^ CD4^+^ T cells with surface expression of CTLA-4 was more decreased in untreated MS patients compared to healthy controls and increased after IFN-β treatment [88].

On the other hand, Pentón-Rol et al. have demonstrated that the in vitro treatment of PBMC from patients with clinically definite RRMS with IFN-α or IFN-β did not show significant differences in the CTLA-4 mRNA levels [89].

Zhou et al. have used a novel model of EAE, an animal model of human MS, to explore if autoimmunity and pathology could depend on B7 co-stimulation by analyzing the in vitro effects of CTLA4-Ig on T cell proliferation, T cell apoptosis, and cytokine production [90]. CTLA4-Ig is a soluble protein that consists of the binding domain of CTLA-4 fused with a Fc portion of IgG, which can interact with B7 molecules and can block the B7:CD28/CTLA-4 pathway [90]. The authors found that the in vitro treatment with CTLA4-Ig could completely block autoreactive T cells [90]. Furthermore, the IL-2 administration was able to reverse the inhibition observed in vitro with CTLA4-Ig [90]. Moreover, the in vitro treatment with CTLA4-Ig decreased the expression of the IL-2 receptor on T cells, enhanced T cell apoptosis, and reduced IL-2, IFN-γ and TNF-α synthesis, whereas it did not affect the synthesis of IL-10 by T cells [90]. Therefore, this study overall suggest that B7-blocking therapies could be a promising potential therapeutic strategy for models of MS [90]. 

### 2.2. In Vivo Studies

Several in vivo studies have explored the role of CTLA-4 in MS, and we have provided a schematic overview of the most important findings from these studies in Table 2.

Before delving into the in vivo study, it is worth mentioning that the EAE model is widely used to study MS. In general, EAE is induced by the immunization of laboratory animals. Usually myelin or its encephalitogenic peptides, as well as recombinant proteins, mixed with adjuvant in order to increase the immune response, are injected into the animal, and after 10-17 days, the first signs of neurological disease should appear [111]. According to the type of model used, it must be remembered that even though a model can be used to study the autoimmunity of the disease, it will not present demyelination in some cases. Moreover, in the case of a chronic model, one has to be careful when evaluating the correct aspects of the disease, since the chronic model has no remission [111].

Almolda et al. have shown that during the recovery phase in an acute EAE female rat model, CTLA-4 expression increased, thus suggesting that it might power the end of the inflammatory/immune response [91].

Cross et al. have studied the effects of a recombinant fusion protein constituted by the extracellular domain of human CTLA-4 bound to mouse IgG2a Fc (CTLA-4-Fc) in a female mouse EAE model [92]. They found that CTLA-4-Fc was able to prevent EAE in 26 out of 28 CTLA-4-Fc-treated mice [92]. Moreover, it was shown that reduced inflammation and nearly no demyelination or axonal loss was recorded in CTLA-4-Fc-treated mice in comparison to the controls [92].

Moreover, Cross et al., exploring the effects of treatment with either CTLA-4-Fc or control Ig in female mice with established EAE, have found that there was a significant improvement in the degree of recovery after the acute episode and after EAE relapses in mice treated with CTLA-4-Fc [93]. Moreover, full clinical remissions were twice as frequent in mice of the CTLA-4-Fc group compared to mice of the placebo groups [93]. However, no effect of CTLA-4-Fc on the relapse rate was recorded [93].

Perrin and colleagues have used CTLA4-Ig, a fusion protein ligand for B7-1 and B7-2, to investigate the role of B7-mediated co-stimulation in chronic relapsing EAE induced by the transfer of MBP specific T cell lines [94]. They have shown that in adoptively transferred EAE, the administration of CTLA-4Ig to donor mice or in the course of the in vitro activation of MBP specific-T cells caused a reduction in clinical disease; conversely, the CTLA4-Ig treatment of recipient animals after the transfer of MBP-activated T cells did not influence the course or severity of the disease [94]. 

In addition, Croxford et al. have found that CTLA4-Ig fusion proteins (CTLA4-Ig) directly delivered into the CNS after EAE induction inhibited disease [95]. They observed that the systemic administration of mouse CTLA4-Ig might inhibit the progression of effector immune responses when administered briefly prior to or in the course of clinical disease and that these were significantly more potent when delivered directly into the CNS [95]. Despite the fact that mouse CTLA4-human Ig was therapeutically less effective than mouse CTLA4-mouse Ig protein, the CTLA4-human Ig gene delivery into the CNS via a non-replicating adenoviral vector was more efficient than a single injection of CTLA4-human Ig protein [95]. Interestingly, gene delivery significantly attenuated EAE development without unavoidably blocking unrelated peripheral immune responses [95]. 

In addition, Khoury et al. have demonstrated that the systemic administration of CTLA4-Ig inhibited clinical disease in a *Lewis* rat model of EAE by suppressing the inflammatory response through the inhibition of Th1 and sparing the Th2 cytokines in the CNS, thus supporting the hypothesis that the blockade of the CD28-B7 T cell costimulatory pathway could protect against active disease by generating a state of immune deviation towards Th2 function [96]. 

Vogel et al. have explored the effect of a B7 blockade in a female mouse EAE model by injecting CTLA4-Ig at days 7 and 9 after immunization [97]. They found that the B7 blockade aggravated disease symptoms and led to a more severe CNS inflammation and demyelination, and was associated with an increased IL-17 and IFN-γ production [97]. Of note, CTLA-4Ig treatment caused a transient reduction of Ki67 and CTLA-4 expression and the function of peripheral Treg cells [97]. Overall, this study suggested that a B7 blockade at a specific stage of the autoimmune response could lead to the suppression of Treg cells, thus causing a more severe disease [97].

Alternatively, Kuchroo et al. have studied the use of anti-B7 antibodies both in vitro and in vivo in EAE [98]. They found that anti-B7-1 decreased the incidence of disease, whereas anti-B7-2 augmented the severity of disease [98]. Moreover, the administration of anti-B7-1 at immunization led to a predominant generation of Th2 clones whose transfer could prevent EAE induction in female animals and could abrogate the established disease [98]. These data suggest that the interaction of B7-1 and B7-2 with shared counterreceptors CD28 and CTLA-4 lead to different results in clinical disease by affecting the commitment of precursors to a Th1 or Th2 lineage [98].

Interestingly, Hurwitz et al. have demonstrated that CTLA-4 engagement can control disease susceptibility in a mouse strain reputed resistant to EAE induction named *BALB/c* [99]. Even though the immunization of the female *BALB/c* mice with syngeneic spinal cord homogenate or an I-A^d^-binding myelin peptide antigen did not cause EAE, an immunization with antigen preparation along with anti-CTLA-4 caused clinical and histological EAE [99]. Moreover, the blockade of CTLA-4 caused a rise in the frequency of antigen-specific T cells that secrete IFN-γ, thus suggesting that CTLA-4 can regulate susceptibility in *BALB/c* mice and might contribute to limiting the expansion of autoreactive T cells and to regulating autoimmune responses [99].

Hurwitz et al., Karandikar et al. and Perrin et al. have demonstrated that the administration of anti-CTLA-4 antibodies accelerated and exacerbated the clinical course of the EAE in female mice [100,101,102]. On the basis of these data, CTLA-4 seems to modulate the intensity of the autoimmune response in EAE, contributing to reducing inflammatory cytokine production and clinical symptoms [102].

Furthermore, Karandikar et al. have demonstrated that the administration of anti-CTLA-4 mAb at different points during the relapsing experimental autoimmune encephalomyelitis (R-EAE) progression in female SJL mice aggravated the clinical disease and increased the T cell reactivity to inducing and relapse-associated epitopes [103]. In addition, they found that the CTLA-4 blockade in the acute disease hindered clinical remission [103].

However, other more recent studies have found that *CTLA-4*–deficiency could effectively protect against EAE in mouse models [104,105,106]. In particular, Verhagen and colleagues have investigated the role of CTLA-4 in EAE by developing a *CTLA*-4KO mouse in which >90% of all CD4+ T cells bear a Vβ8.2 transgenic T-cell receptor, specific for myelin basic protein peptide Ac1–9 (ASQKRPSQR), and they found that these mice, belonging to both genders, do not develop EAE and are resistant to disease induction [104]. 

Moreover, Paterson and colleagues have conditionally ablated *CTLA-4* in adult mice and they have demonstrated—conversely to the germline *CTLA-4* deficiency, which can be lethal—that *CTLA-4* deletion in adult mice protected against EAE [105]. 

Alternatively, Klocke et al. have found that *CTLA-4* deficient adult mice were protected against peptide-induced EAE but they were not protected against protein-induced EAE, although the onset of protein-induced EAE was significantly delayed [106].

Another interesting approach was proposed by Lim and colleagues, who have identified a cell-permeable peptide (dNP2) able to effectively deliver proteins both in mouse and human T cells and different tissues and penetrate the brain tissue and resident cells via blood vessels passing through the blood–brain barrier [107]. Noteworthily, they have shown that the dNP2-conjugated cytoplasmic domain of cytotoxic T-lymphocyte antigen 4 (dNP2-ctCTLA-4) could negatively regulate activated T cells and exert an inhibitory effects in preventive and therapeutic mouse models of EAE, decreasing demyelination and the CNS-infiltrating T helper 1 and T helper 17 cells. This experiment was carried out using both male and female animals [107]. Hence, using dNP2 to deliver ctCTLA-4 into CNS-infiltrating T cells might be considered a novel potentially effective therapeutic approach to MS [107].

Moreover, Kim et al. have shown that the synthetic chimeric peptide dNP2-ctCTLA-4 induced Foxp3^+^ Tregs in mouse and human PBMC, and it can attenuate EAE progression with long-term regulation and prevent relapse [108]. In particular, dNP2-ctCTLA-4 can control TGF-β signaling to augment Foxp3 expression during the Th1 or Th17 differentiation and expression of functional molecules, including CTLA-4 [108]. Therefore, this peptide seems to be an interesting potential drug to raise the number of Tregs in autoimmune disorders, including MS. Female animals were used for the experiments [108]. 

Another possible strategy was suggested by Spanier et al., who have recently demonstrated the supplementation of Vitamin D3 before EAE induction raised the expression of CTLA-4 by spinal cord-infiltrating CD4+ Tconv and Treg cells, reduced the EAE incidence, and diminished clinical severity only when there was a functional Cyp27b1 gene in the activated microglial cells and macrophages [109]. Overall, this study suggested that CTLA-4 could act as a vitamin D_3_-regulated immunological checkpoint in the prevention of MS. Female mice were used for the experiments [109].

Moreover, Kim et al. have identified another interesting approach [110]. They synthesized an AP-ctCTLA-4 peptide to modulate the T cell function in a mouse model of EAE, and they showed that AP-ctCTLA-4 decreased IL-17A expression under Th17 differentiation conditions in vitro and improved EAE, with reduced numbers of pathogenic IL-17A^+^GM-CSF^+^ CD4 T cells. Both male and female animals were used for the experimental procedures [110]. 

## 3. Clinical studies

### 3.1. Genetic Studies

Considering that CTLA-4 is a critical negative regulator of T-cell function and is involved in the mechanism of action of CD4+ CD25+ regulatory cells, the *CTLA-4* gene, located on chromosome 2q33 region, seems to be a very attractive candidate gene with respect to MS susceptibility [112].

Different studies have investigated the possible correlation between several *CTLA-4* polymorphisms and MS with conflicting results (Table 3) [43]. 

Qiu et al. have conducted an association study of different SNPs in non-HLA genes, including *CTLA-4*, in a Western Australian cohort of patients with MS and among controls [113]. They showed that the interactive effects of the *CTLA-4* and *CLEC-16A* polymorphisms were gender-specific and in particular were protective only in females [113].

Alternatively, two European independent family-based studies involving MS family trios conducted by Alizadeh et al. demonstrated the interaction of the *CTLA-4* gene with the DRB1*15 haplotype regarding MS genetic susceptibility [112].

Wagner et al. have analyzed the possible association of different polymorphisms in certain genes, including *CTLA-4*, with MS susceptibility and/or progression [114]. They found that in HLA-DRB1*15:01 negative subjects, the G allele in rs231775A > G of the *CTLA-4* gene was associated with an increased risk of MS [114]. 

Harbo et al. have shown that the *CTLA-4* exon 1 (+49)- heterozygous A-G genotype is significantly higher in Norwegian MS patients in comparison to healthy controls, whereas the *CTLA-4* promoter (ª318) dimorphism is not involved in genetic susceptibility [115]. 

Kantarci et al. have further demonstrated an association between the common multiple loci genotype of *CTLA-4* and a higher susceptibility to MS on the basis of a haplotype analysis in a population-based sample in Olmsted County and on a pooled analysis of Caucasian populations [116].

Ligers et al. have explored whether there was an association between a genetic susceptibility to MS and three intragenic polymorphisms of the *CTLA-4* gene, a C/T base exchange in the promoter (p.-318), an A/G substitution in exon 1 (p.49), and a dinucleotide repeat polymorphism in exon 4 (p.642) [117]. They found a significant association for homozygosity for the G^49^ allele in a case-controlled analysis that compared MS patients and controls, together with a transmission disequilibrium for the G^49^ allele in MS families [117]. Moreover, they found evidence for linkage by the affected pedigree member analysis and a transmission distortion of the exon 4^642^ polymorphism [117]. Overall, this study highlighted that the *CTLA-4* gene could be involved in the genetic susceptibility to MS and suggested that a dysregulation of the *CTLA-4*-driven downregulation of T-cell activation might be implicated in the pathogenesis of MS [117].

Moreover, Ligers et al. have genotyped for *CTLA-4* polymorphisms and have explored the expression by PBMC of CTLA-4 mRNA and protein when comparing MS patients, myasthenia gravis patients, and healthy controls [118]. They found that although the mRNA and protein expression levels were comparable in the patients and controls, there was a relationship between genotype and CTLA-4 expression [118]. In particular, subjects with thymine at position -318 of the *CTLA-4* promoter (T^-318^) and homozygous for adenine at position 49 in exon 1 had an significantly augmented expression of cell-surface CTLA-4 after cellular stimulation and of CTLA-4 mRNA in non-stimulated cells [118]. The association was observed most distinctly for unsorted CD3^+^ cells, whereas it was not present in the CD8^+^ subset [118].

Mkhikian et al. have shed light on the possibility of a unifying molecular mechanism in MS, whereby multiple genetic variants, including *CTLA-4*, could combine with multiple environmental factors, such as sunlight/vitamin D_3_ and metabolism, and could converge to dysregulate Golgi N-glycosylation [119].

Barcellos et al. have shown that a common variant within *CTLA-4* was strongly associated with MS in families with other autoimmune disorders but not in families without other autoimmune diseases [120].

Bilińska et al. have found that the *CTLA-4* (A49G) exon 1 polymorphism is associated with MS progression [121].

Fukazawa and colleagues have studied Japanese patients with and without clinically or radiographically fulminant attacks, which are attack-related clinically or radiologically severe relapses but do not necessarily entail a severe disability, who satisfied the diagnostic criteria of MS and have found that the GG homozygous and G alleles of the *CTLA-4* gene A/G coding SNP at position 49 in exon 1 were significantly more frequent in patients with fulminant attacks in comparison to those without [122]. 

Since an +49A to G transition in exon 1 of *CTLA-4* gene results in an amino acid substitution in the leader peptide and could influence the CTLA-4 expression in T cells, Dinčić et al. have analyzed both the separate and combined effect of *IL-1β* TaqI, *IL-1ra* VNTR, and *CTLA-4* +49 A/G polymorphisms on MS susceptibility, its clinical course, and its progression in a Serbian population [123]. They found a significant independent relative risk for MS susceptibility in noncarriers of *IL-1ra* allele 2 and *CTLA-4* + 49 AA genotype as well as their combined effect, thus suggesting the involvement of *IL-1ra* VNTR and *CTLA-4* A/G + 49 gene polymorphisms in the susceptibility to MS [123].

Suppiah and colleagues have explored the *CTLA-4* +49A/G and CT60 polymorphisms in Flanders MS families [124]. They found that the +49 G-allele was significantly more transmitted to affected probands, whereas there was no transmission distortion for the CT60 polymorphism [124]. Moreover, a significant overtransmission of the +49 A/G*G –CT60*G haplotype and undertransmission of the +49 A/G*A –CT60*G haplotype was observed [124].

Malferrari et al. have analyzed the association of two *CTLA-4* polymorphisms (+49 A/G and -318 C/T) with MS by genotyping Italian sporadic MS patients and healthy controls [125]. Although they found no differences in the allelic and genotypic frequencies between patients and controls with respect to single-loci variations, taking into account a putative interaction at the two loci, it was found that the T/G combination was more commonly recorded in MS patients compared to controls, thus highlighting that this allelic combination of the *CTLA-4* polymorphisms could be implicated in MS susceptibility among Italians [125]. 

Yousefipour et al. have shown preliminary evidence that a *CTLA-4* genetic variation at -1661 locus could contribute to MS susceptibility in Iranians [126]. Moreover, the TACA haplotype could be protective [126].

Heggarty et al. have analyzed the *CTLA-4* −318 C/T promoter SNP, the +49 A/G exon 1 SNP, the 3′ UTR AT_n_ dinucleotide repeat, and the intergenic CT60 polymorphisms in Northern Irish RRMS and PPMS patients and healthy controls [127]. They found that there was an association between the A allele of the exon 1 +49 A/G SNP and the AA genotype with RRMS, but not with PPMS [127]. Moreover, they observed that the allele distribution of the AT_n_ microsatellite in the PPMS population was significantly different from the controls [127]. Therefore, *CTLA-4* + 49 A/G and 3′UTR polymorphisms might be potential modifiers of the disease course in MS [127]. 

Masterman and colleagues have conducted a two-stage study in order to analyze the role of *CTLA-4* promoter–exon 1 haplotypes in the age at onset, disease severity, and disease course in MS [128]. In stage one, deviations in the *CTLA-4* haplotype frequencies were found in patients sub-grouped according to disease course, whereas in stage two, none of these associations were found [128]. 

Interestingly, Mäurer et al. have analyzed the association of the *CTLA-4* A/G dimorphism in exon 1 (+ 49) with MS susceptibility, course, and severity [129]. They found that although there was no differences in the allelic distribution of the G^49^ allele between the MS patients and the controls, the G^49^ allele was present in a significantly larger percentage of PPMS patients in comparison to patients with a bout onset of the disease, thus supporting the hypothesis that *CTLA-4* mutations could be involved in the pathogenesis of PPMS by affecting the down regulation of T cell function and thus contributing to the low-grade inflammatory process that characterizes PPMS [129].

Fransen et al. have investigated whether SNPs that correlate with a clinical MS course could correlate with specific MS lesion characteristics in autopsy tissue by genotyping MS brain donors from the Netherlands Brain Bank MS autopsy cohort [130]. Interestingly, among the genetic variants that were discovered to have an effect on post-mortem MS lesion characteristics, it was found that rs5742909/*CTLA-4* was associated with the proportion of remyelinated lesions [130].

Karabon et al. have studied the association of the *CTLA-4* gene polymorphisms −319C/T, +49A/G, (AT)_n_, CT60A/G, and Jo31G/T with the levels of both membrane CTLA-4 (mCTLA-4) and cytoplasmic CTLA-4 (cCTLA-4) in the CD4^+^ T lymphocytes of MS patients and with MS susceptibility and clinical course [131]. They found that there was an association between the Jo31GG and CT60GG genotypes and a reduced mean fluorescence intensity (MFI) of the total CTLA-4 (mCTLA-4 + cCTLA-4) molecules in CD4^+^ T cells from RRMS and SPMS patients in comparison with others, thus suggesting that the presence of the Jo31G allele and/or of the CT60G allele was associated with MS susceptibility [131]. Moreover, the percentages of cells that express mCTLA-4 and cCTLA-4 in RRMS patients were increased in the carriers of the alleles non-predisposed to MS (CT60A and Jo31T); however, the percentages of corresponding cells were more decreased in SPMS patients than in RRMS patients [131]. Moreover, a higher risk of paresthesia and pyramidal signs as first symptoms of the disease and an earlier transition to the SP form in those patients have also been shown [131]. Overall, these data supported the hypotheses that the reducing frequencies of cells that expressed immunosuppressive mCTLA-4 and cCTLA-4 in carriers of alleles non-predisposed to MS could result in an inappropriate down-regulation of the ongoing T-cell responses in these patients and consequently cause the earlier disease progression from the RR to the SP form [131].

On the other hand, Čizmarević et al. have studied the possible role of *CTLA-4* +49 A/G gene polymorphism in MS susceptibility and disease behavior in Croatian and Slovenian populations and found no significant differences in the *CTLA-4* +49 A or G allele distribution between MS patients and the controls, thus suggesting that this polymorphism is not involved in MS susceptibility in the surveyed populations [132]. 

Dyment et al. have genotyped two polymorphisms of the *CTLA-4* gene (a 3’ untranslated (AT)_(n)_ microsatellite and an alanine/threonine RFLP of exon 1) in sibling pairs with MS and they found that there was no evidence for linkage by either identity-by-descent (ibd) or identity-by-state (ibs) methods and that there was no preferential transmission of alleles [133]. In addition, after the stratification of the patients, there was no preferential transmission according to gender, the presence or absence of HLA*DRB1*15, ethnicity, or clinical course [133]. Overall, *CTLA-4* does not seem to be a significant MS susceptibility locus in Canadian multiplex families [133]. 

In addition, Roxburgh and colleagues have analyzed the *CTLA-4* gene variation in a wide cohort of MS trio families (an affected individual and both parents) from the United Kingdom by genotyping them for the 3’ untranslated region variable number tandem repeat, the CT60 single nucleotide polymorphism (SNP), and five haplotype-tagging SNPs [134]. This study showed no evidence of an association between any individual marker or common haplotypes and the disease, thus suggesting that any effect of *CTLA-4* on MS susceptibility should probably be very small [134].

Moreover, Greve et al. have performed a case-control association study in German, Hungarian, and Polish MS patients and a group of controls to investigate the role of *CTLA-4* CT60 and +49A/G polymorphisms [135]. They have shown that there was no consistent association of these polymorphisms or respective haplotypes with MS and that there was no association of CT60 genotypes with T cell expression of ICOS and CTLA-4 after in vitro stimulation [135]. 

Fukazawa and colleagues have found that the *CTLA-4* exon 1 polymorphism was similar when comparing Japanese MS patients and controls; however, this study has shown that this *CTLA-4* polymorphism could modulate the prognosis of MS patients [136].

The same group of authors then analyzed the polymorphisms of exon 1 (+49A/G) and the promoter (-318C/T and -651C/T) regions of the *CTLA-4* gene in Japanese MS patients and healthy controls and they found that there were no significant differences in the distribution of polymorphisms between the two groups and that there was no association between the clinical characteristics and the polymorphisms [137].

A study conducted by Borhani Haghighi et al. in Iranian MS patients has shown the lack of significant associations between the *CTLA-4* exon 1 polymorphism and MS nor any of its subtypes [138].

Furthermore, Heidari et al. have analyzed three single nucleotide polymorphisms (SNPs) (-318C/T, +49A/G, +6230A/G) of the *CTLA-4* gene in RRMS patients and healthy controls [139]. They found that although the genotypes -318 CC and +49 AA were overrepresented in MS patients, these differences were not statistically significant, thus suggesting that there was no significant association with the alleles and genotypes of SNPs of *CTLA-4* in Iranian MS patients [139]. 

In addition, Luomala et al. have shown that the *CTLA-4* polymorphism does not play a significant role in MS development in the Finnish population [140].

According to these results, Van Veen et al. have shown that the *CTLA-4*-318, *CTLA-4* + 49, and *CD28*-I3 + 17 polymorphisms were not associated with the risk of developing MS and did not change the course of the disease [141]. 

Moreover, Wray and colleagues have demonstrated that there was no significant association between the *CTLA-4* A49G genotype and the risk of MS independently of the DR15 status in southern Australians [142].

In addition, Boćko et al. showed that the distribution of *CTLA-4* exon 1 A(49)G genotype, phenotype, and allele frequencies was not different when comparing healthy controls with unrelated Polish MS patients in the Lower Silesia region [143].

Rasmussen et al. have explored the possibility for a genetic association between *CTLA-4* and MS in European Caucasians and in individuals of Shanghai-Chinese origin and they also investigated if a genetic interaction between *CTLA-4* and HLA-DR2 could be involved in MS susceptibility [144]. They found that there were no significant differences in the distribution of genotypes or haplotypes of the *CTLA-4* gene between the patients with MS and controls in the two populations [144]. Moreover, there was no evidence indicating the involvement of the interaction between HLA-DR2 and *CTLA-4* in MS development among European Caucasians; conversely, the analysis of the Shanghai Chinese subjects indicated the presence of such an interaction [144].

Moreover, a study conducted by Lorentzen et al. did not confirm an association of the *CD28*/*CTLA-4*/*ICOS* gene region in a Norwegian MS cohort [145].

Teutsch et al., exploring the associations between MS and polymorphisms in the *CTLA-4* gene in Australian patients, showed that there was no significant association between MS and the *CTLA-4* exon 1 +49 alleles [146]. In addition, a meta-analysis demonstrated no significant association across nine comparable datasets nor with PPMS across seven datasets [146]. Moreover, a haplotype analysis has shown a trend towards a reduction in the *CTLA-4*-1722C, -1577G, and +49G haplotypes in +49 G positive patients with MS in comparison with the controls [146].

Bagos et al. performed a meta-analysis regarding the association of the risk of MS and the *CTLA-4* gene polymorphisms and in particular exploring the exon 1 +49 A/G and the promoter -318 C/T polymorphisms [147]. This meta-analysis showed no evidence of an association of the two polymorphisms of *CTLA-4* and MS [147].

Liu et al. have conducted a comprehensive meta-analysis study in order to evaluate the association between *CTLA-4* and the risk of MS, thus clarifying the conflicting results from the individual studies in this field, and they found that 49A/G, 318C/T, or CT60A/G polymorphisms, both in the total analysis and in subgroup analyses, were not found to be significantly associated with MS [148]. 

Furthermore, a meta-analysis conducted by Haibing et al. showed that there were no significant associations between the *CTLA-4* gene rs221775A>G single nucleotide polymorphism and MS susceptibility for a dominant genetic model, a homozygous genetic model and a recessive genetic model [149].

According to this line of research, a meta-analysis performed by Song et al. found that there were no associations between the *CTLA-4* +49 A/G and -318 C/T polymorphisms and MS susceptibility in Caucasians, Asians, and Arabs [150].

Although Palacios et al. have found a reduction in the expression of *CTLA-4* isoforms associated with certain alleles of the SNP −658 in MS patients and not in controls, which suggested the occurrence of epigenetic changes generated by the disease process, their overall suggestion was that the role of CTLA-4 in the pathogenesis of MS might be associated with the functional changes in its pathway rather than to genetic polymorphisms [151]. 

Overall, the controversial results from these studies might be due to differences between the population samples, ethnic backgrounds, and selection criteria of the patients, and it should also be noted that disease heterogeneity can influence the involvement and nature of genetic susceptibility factors [139]. Hence, further studies are needed to explore this field, involving larger populations and analyzing the possible link between different clinical subtypes and the role of *CTLA-4* polymorphisms [139].

### 3.2. Other Clinical Studies 

Several other clinical studies in this field have been conducted, including studies aimed to investigate the effects of common MS therapies on CTLA-4 and case reports (Table 4).

#### 3.2.1. Clinical Studies Investigating the Effects of Common MS Therapies on CTLA-4

Espejo et al. have investigated the effects of IFN-β on the CD28/CTLA-4:CD80/CD86 costimulatory signal pathway, comparing RRMS patients before and 3 months after starting IFN-β treatment and healthy controls [152]. They found that 3 months of in vivo IFN-β treatment did not modulate the T-lymphocyte proliferative response via the CD28/CTLA-4 pathway [152]. Nonetheless, after 3 months of in vivo IFN-β treatment, the CD28/CTLA-4-mediated pathway was changed via the reduction of CD80-induced IL-2 production [152]. Since the production of IL-2 is needed for lymphocyte activation and the development of the autoimmune response, these data could indicate that a possible immunomodulatory effect of IFN-β treatment in RRMS could be a limitation of the autoimmune response, thus modifying the CD80:CD28/CTLA-4 pathway [152].

#### 3.2.2. Case Reports

A case report described by Lin and colleagues has shown that a patient with MS and immune dysregulation had a heterozygous mutation in *CTLA-4*, and had an excellent clinical response to abatacept [153].

Kaninia et al. described a case of biopsy-proven CNS inflammatory demyelination occurring in the context of primary immunodeficiency and of a new *CTLA-4* variant [154]. This report sustained the role of CTLA-4 as a regulator of T-cell activation and immune tolerance and highlighted that CTLA-4 pathway alteration can lead to inflammatory demyelination, thus having significant implications for the development of new possible treatments for autoimmune disorders such as MS [154].

### 3.3. Clinical Trials

Several clinical trials have investigated the possibility of using CTLA4-Ig in MS (Table 5) [77,78]. 

Viglietta and colleagues have conducted a phase 1 open-label, dose-escalation clinical trial in order to evaluate the safety and tolerability of CTLA4-Ig infusion and its action towards immune function in RRMS patients [77]. CTLA4-Ig was manufactured as RG2077, a recombinant CTLA4-IgG4m fused to the heavy-chain constant region of the human IgG4 isotype [77]. In this study, 16 RRMS patients were treated with a single CTLA4-Ig infusion and then followed up for up to 3 months [77]. Furthermore, four additional participants were involved in an extension study and were treated with four doses of CTLA4-Ig and monitored for 6 months [77]. This study showed that CTLA4-Ig was well tolerated in MS patients and the majority of adverse events were classified as mild [77]. Moreover, a decrease in MBP proliferation within 2 months of infusion and a reduction in IFN-γ production by MBP-specific lines were shown [77]. Overall, the selective blockade of the CD28–B7 costimulatory pathway using CTLA4-Ig appears to be safe and well tolerated in MS patients and could be a promising strategy for controlling T-cell activity and inflammation in MS [77].

The phase II clinical trial NCT00035529 was conducted to evaluate whether abatacept could reduce MS disease activity upon MRI examinations and whether it could reduce the rate of clinical MS exacerbations in comparison to a placebo in RRMS patients. This trial was terminated early because of safety hazards; nevertheless, the results of this study were uncertain owing to the imbalance in disease activity among the treatment groups at the baseline [78].

Moreover, the phase II, randomized, double-blind, placebo-controlled, and multi-center trial ACCLAIM (A Cooperative Clinical Study of Abatacept in Multiple Sclerosis, NCT01116427) conducted by Khoury and colleagues has assessed the efficacy and safety of abatacept versus placebo in RRMS patients [78]. A total of 65 of the 123 projected RRMS patients involved in this study were randomly divided to receive monthly intravenous infusions of abatacept or a placebo for a period of 24 weeks in a 2:1 ratio; then, they were switched to the opposed treatment at 28 weeks, and were treated with their final dose of the study medication at 52 weeks [78]. However, the enrollment was concluded prematurely because of a slow accrual [78]. Although abatacept was found to be well tolerated, it was demonstrated that there were neither significant differences in the number of new gadolinium-enhancing (Gd+) MRI lesions when comparing the abatacept and placebo groups, nor significant differences in any of the other MRI and clinical parameters of the disease activity [78]. Even though this study did not show the efficacy of abatacept in RRMS patients, its potential efficacy in wider studies with participants with higher disease activity cannot be excluded [78].

Moreover, Glatigny et al. have analyzed the specimens from the ACCLAIM trial to investigate the effects of abatacept on the frequency and transcriptional profile of specific T cell populations in peripheral blood [79]. They discovered that the relative abundance of CD4+T follicular helper (Tfh) cells and regulatory T cells was selectively reduced in participants after treatment with abatacept [79]. Moreover, they found that in both cell types, the abatacept treatment decreased the proportion of activated cells that express CD38 and ICOS and was associated with the reduced expression of genes involved in cell division and chromatin dynamics; after the end of the abatacept treatment, the cellular and molecular alterations were reversed [79].

## 4. Conclusions

**Figure 2:**
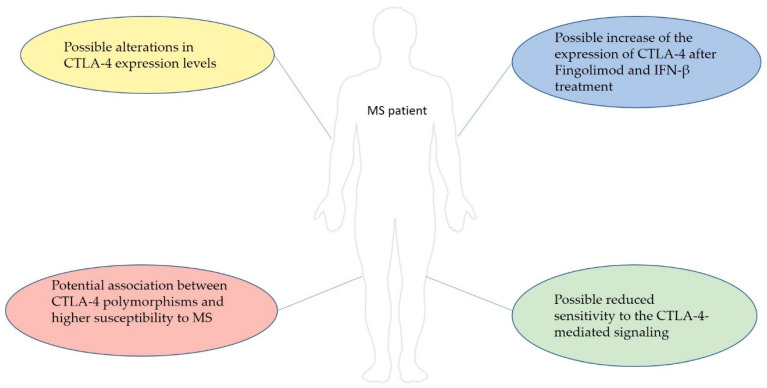
Overview of the main data suggesting a possible role of CTLA-4 in the pathogenesis of MS and its possible involvement in the mechanism of action of common MS therapies. The results show the importance of CTLA-4 levels in the developing of MS, highlighting a general picture where a decreased presence of CTLA-4 might be detrimental for protection from MS. This evidence could be the basis for future therapeutic approaches.

OverNumerous studies have investigated the possible role of CTLA-4 in the pathogenesis of MS and the potential therapeutic effects of its modulation, revealing many interesting but often conflicting data (Figure 2). The decreased level of CTLA-4 in PBMC of patients with MS proved its importance in the development of the disease, indicating itself as a valid candidate for therapies. Even though animal models confirmed this evidence, demonstrating how CTLA-4-like molecules can ameliorate EAE symptoms, there are not many clinical trials that assure the efficacy of the only mAb currently available: abatacept. Attempting to associate genetic *CTLA-4* polymorphisms to MS seemed difficult due to the high genetic variability in the population between different country, making it difficult to draw a single picture regarding how *CTLA-4* mutations can modulate the onset of MS.

Since genetic correlation does not seem to be the correct pathway due to the many conflicts in the results, further studies are needed to reveal the exact mechanisms underlying the action of this crucial immune checkpoint. This may allow for the refinement of the molecules used for human treatment along with the associated clinical protocols, thereby potentially bridging the gap between in vivo evidence and clinical trials and possibly leading to the identification of novel potential immunotherapeutic strategies for MS patients.

## Figures and Tables

**Table 1:** Schematic overview of the most important evidence from the in silico, in vitro, and ex vivo studies investigating the role of CTLA-4 in MS.

In Silico, in Vitro, and Ex Vivo Studies	
Evidence	References
- Similar expression of CTLA-4 on CD4+ and CD8+ T cells between the treated and untreated MS patients - No significant differences in the expression of CTLA-4 on monocytes or CD4+ and CD8+ T cells between the treated or untreated MS patients compared with healthy controls - Raised expression of CTLA-4 on both T-cell subsets in peripheral blood mononuclear cells (PBMC) of an MS patient with a very rapidly progressing disease	[80]
- CTLA-4 levels not statistically significantly different in PBMC from healthy controls and patients with untreated MS	[81]
- No significant differences in the CTLA-4 expression levels on T cells in PBMC from MS patients in comparison with healthy subjects - The blockade of CTLA-4-mediated signaling during the stimulation of MBP-reactive T cells from healthy subjects increased the proliferative and cytokine responses; blocking CTLA-4 in MS patients had fewer effects	[76]
- Decreased expression of CTLA-4 in RRMS patients compared with controls	[82]
- Decreased levels of sCTLA-4 in MS patients in comparison to controls - sCTLA-4 did not correlate with EDSS score in MS and NMO patients	[83]
- Strong age-dependent decrease in the expression of CTLA-4 on memory CD8 T cells in healthy donors and not in MS patients	[84]
- Increased median percentage of freshly isolated peripheral blood CD4+/CTLA-4+ T cells from MS patients	[85]
- Reduced CTLA-4 expression in naïve patients compared to healthy subjects - Several treatments (e.g., fingolimod) can induce the expression of CTLA-4	[86]
- Interferon (IFN)-β augmented the expression of the CTLA-4 intracellular molecules	[87]
- Increased percentage of CD4+CD25^high^ T cells with total (intracellular + surface) expression of CTLA-4 in MS patients in comparison to controls, independently of IFN-β treatment - Decreased percentage of CD25^high^ CD4+ T cells with surface expression of CTLA-4 in untreated MS patients compared to healthy controls, which was raised after IFN-β treatment	[88]
- In vitro treatment of PBMC from RRMS patients with IFN-α or IFN-β did not show significant differences in the CTLA-4 mRNA levels	[89]
- In vitro treatment with CTLA4-Ig could completely block autoreactive T cells	[90]

**Table 2:** Schematic overview of the most important evidence from the preclinical in vivo studies investigating the role of CTLA-4 in MS.

In Vivo Studies	
Evidence	References
- CTLA-4 expression increased during the recovery phase in an acute experimental autoimmune encephalomyelitis (EAE) model	[91]
- CTLA-4-Fc prevented EAE in 26/28 CTLA-4-Fc-treated mice - There was reduced inflammation and nearly no demyelination or axonal loss in CTLA-4-Fc-treated mice in comparison to controls	[92]
- Significant improvement in the degree of recovery after an acute episode and after EAE relapses in mice treated with CTLA-4-Fc - Full clinical remission is twice as frequent in mice from the CTLA-4-Fc group as in mice from the placebo groups [93] - No effect of CTLA-4-Fc on relapse rate	[93]
- In adoptively transferred EAE, the administration of CTLA-4Ig to donor mice or in the course of in vitro activation of MBP specific-T cells caused reduction of clinical disease - CTLA4-Ig treatment of recipient animals after the transfer of MBP-activated T cells did not influence the course and severity of the disease	[94]
- CTLA4-Ig directly delivered in the central nervous system (CNS) after EAE induction inhibited the disease	[95]
- The systemic administration of CTLA4-Ig inhibited clinical disease in a model of EAE	[96]
- B7 blockade in an EAE model by CTLA4-Ig aggravated disease symptoms and led to more severe CNS inflammation and demyelination	[97]
- Anti-B7-1 antibodies decreased the incidence of disease in EAE models - Anti-B7-2 antibodies augmented the severity of disease in EAE models	[98]
- CTLA-4 engagement can control disease susceptibility in a mouse strain resistant to EAE induction	[99]
- Anti-CTLA-4 antibodies accelerated and exacerbated the clinical course of the EAE	[100,101,102]
- CTLA-4 blockade during acute disease hindered clinical remission in an R-EAE model	[103]
- *CTLA-4*–deficiency could protect from EAE in mouse models	[104,105,106]
- dNP2-ctCTLA-4 could negatively regulate activated T cells and exerted inhibitory effects in preventive and therapeutic models of EAE	[107]
- dNP2-ctCTLA-4 can attenuate EAE progression with long-term regulation and prevent relapse	[108]
- CTLA-4 could act as a vitamin D_3_-regulated immunological checkpoint in the prevention of MS	[109]
- AP-ctCTLA-4 improved EAE	[110]

**Table 3:** Schematic overview of the most important evidence from the clinical genetic studies investigating the role of CTLA-4 in MS.

Genetic Studies	
Evidence	References
- The interactive effects of the *CTLA-4* and *CLEC-16A* polymorphisms were gender-specific and protective only in females	[113]
- Interaction of the *CTLA-4* gene with the DRB1*15 haplotype in MS genetic susceptibility	[112]
- In HLA-DRB1*15:01 negative subjects, G allele in rs231775A > G of *CTLA-4* gene was associated with an increased risk of MS	[114]
- The *CTLA-4* exon 1 (+49)- heterozygous A-G genotype is significantly higher in MS patients in comparison to healthy controls - The *CTLA-4* promoter (ª318) dimorphism is not involved in MS genetic susceptibility	[115]
- There is an association between the common multiple loci genotype of *CTLA-4* and higher susceptibility to MS	[116]
- An association was found regarding homozygosity for the G^49^ allele in a case-control analysis that compared MS patients and controls - Transmission disequilibrium for the G^49^ allele in MS families	[117]
- Subjects with thymine at position -318 of the *CTLA-4* promoter (T-^318^) and homozygous for adenine at position 49 in exon 1 had significantly augmented expression of cell-surface CTLA-4 after cellular stimulation and of CTLA-4 mRNA in non-stimulated cells	[118]
- Possible molecular mechanism in MS: multiple genetic variants, including *CTLA-4*, could combine with multiple environmental factors, (e.g., sunlight/vitamin D_3_ and metabolism), and could converge to dysregulate Golgi N-glycosylation	[119]
- A common variant within *CTLA-4* was strongly associated with MS in families with other autoimmune disorders but not in families without other autoimmune diseases	[120]
- The *CTLA-4* (A49G) exon 1 polymorphism is associated with MS progression	[121]
- The GG homozygous and G alleles of the *CTLA-4* gene A/G coding SNP at position 49 in exon 1 were significantly more frequent in patients with fulminant attacks in comparison to those without	[122]
- Possible involvement of *IL-1ra* VNTR and *CTLA-4* A/G + 49 gene polymorphisms in susceptibility to MS	[123]
- The +49 G-allele was significantly more transmitted to affected probands; there was no transmission distortion for the CT60 polymorphism - Significant over-transmission of the +49 A/G*G – CT60*G haplotype and under-transmission of the +49 A/G*A –CT60*G haplotype	[124]
- Association of two *CTLA-4* polymorphisms (+49 A/G and −318 C/T) with MS	[125]
- Preliminary evidence that CTLA-4 genetic variation at -1661 locus could contribute to MS susceptibility - The TACA haplotype could be protective	[126]
- Association between the A allele of the exon 1 +49 A/G SNP and the AA genotype with RRMS, but not with PPMS - The allele distribution of the ATn microsatellite in the PPMS population was significantly different from controls	[127]
- Two-stage study: - Stage 1: deviations in *CTLA-4* haplotype frequencies were found in patients subgrouped according to disease course - Stage 2: none of these associations were found	[128]
- No differences in the allelic distribution of the G^49^ allele between MS patients and controls - The G^49^ allele was present in a significant larger percentage of PPMS patients in comparison to patients with bout onset of disease	[129]
- rs5742909/*CTLA-4* was associated with the proportion of remyelinated lesions	[130]
- Association between the Jo31GG and CT60GG genotypes and reduced MFI of total CTLA-4 (mCTLA-4 + cCTLA-4) molecules in CD4^+^ T cells from RRMS and SPMS patients - The presence of the Jo31G allele and/or of the CT60G allele can be associated with MS susceptibility - The percentages of cells which express mCTLA-4 and cCTLA-4 in RRMS patients were increased in carriers of the alleles non-predisposing to MS (CT60A and Jo31T)	[131]
- No significant differences in *CTLA-4* +49 A or G allele distribution between MS patients and controls	[132]
- *CTLA-4* does not seem to be a significant MS susceptibility locus	[133]
- Any effect of *CTLA-4* on MS susceptibility should probably be very small	[134]
- No consistent association of *CTLA-4* CT60 and +49A/G polymorphisms or respective haplotypes with MS - No association of CT60 genotypes with T cell expression of ICOS and CTLA-4 after in vitro stimulation	[135]
- *CTLA-4* exon 1 polymorphism was similar when comparing MS patients and controls - This *CTLA-4* polymorphism could modulate the prognosis of MS patients	[136]
- No significant differences in the distribution of *CTLA-4* polymorphisms between MS patients and healthy controls - No association between clinical characteristics and the polymorphisms	[137]
- Lack of significant associations between *CTLA-4* exon 1 polymorphism and MS	[138]
- No significant association with alleles and genotypes of SNPs of *CTLA-4* in MS patients	[139]
- *CTLA-4* polymorphism did not play a significant role in MS development	[140]
- *CTLA-4*-318, *CTLA-4* + 49 and *CD28*-I3 + 17 polymorphisms were not associated with the risk of developing MS and did not change the course of disease	[141]
- No significant association between the *CTLA-4* A49G genotype and the risk of MS independently of the DR15 status	[142]
- The distribution of *CTLA-4* exon 1 A(49)G genotype, phenotype, and allele frequencies was not different when comparing healthy controls with unrelated MS patients	[143]
- No significant differences in the distribution of genotypes or haplotypes of the *CTLA-4* gene between patients with MS and controls	[144]
- No association of the* CD28/CTLA-4/ICOS* gene region in a MS cohort	[145]
- No significant association between MS and the *CTLA-4* exon 1 +49 alleles - trend towards a reduction of the *CTLA-4*-1722C, −1577G, +49G haplotype in +49 G positive patients with MS in comparison with controls	[146]
- No evidence for association of two polymorphisms of *CTLA-4* (exon 1 +49 A/G polymorphism and promoter −318 C/T polymorphism) and MS	[147]
- 49A/G, 318C/T, CT60A/G polymorphism: not significantly associated with MS	[148]
- No significant association between *CTLA-4* gene rs221775A>G single nucleotide polymorphism and MS susceptibility	[149]
- No associations between the *CTLA-4* +49 A/G and -318 C/T polymorphisms and MS susceptibility	[150]
- Reduction in the expression of *CTLA-4* isoforms associated with certain alleles of SNP − 658 in MS patients	[151]

**Table 4:** Schematic overview of the most important evidence from the clinical studies investigating the effects of common MS therapies on CTLA-4 and from the case reports on CTLA-4 in MS.

Clinical Studies Investigating the Effects of Common MS Therapies on CTLA-4	
Evidence	References
- Three months of in vivo IFN-β treatment did not modulate the T-lymphocyte proliferative response via the CD28/CTLA-4 pathway in RRMS patients - After 3 months of in vivo IFN-β treatment, the CD28/CTLA-4-mediated pathway was changed via the reduction of CD80-induced IL-2 production	[152]
**Case reports**	
Evidence	References
- A patient with MS and immune dysregulation with a heterozygous mutation in *CTLA-4* had an excellent clinical response to abatacept	[153]
- A case of biopsy-proven CNS inflammatory demyelination in the context of primary immunodeficiency and of a new *CTLA-4* variant - CTLA-4 pathway alteration can lead to inflammatory demyelination	[154]

**Table 5:** Clinical trials investigating the possibility of using CTLA4-Ig in MS.

ClinicalTrials.gov Identifier	Official Title	Recruitment Status	Intervention/Treatment	Phase
NCT00076934	A Phase I Study: Safety of RG2077 (CTLA4-IgG4m) in Patients with Relapsing-Remitting Multiple Sclerosis	Completed	Drug: RG2077 (CTLA4-IgG4m)	Phase 1
NCT00035529	A Phase II, Randomized, Double-Blind, Placebo Controlled Study to Evaluate the Preliminary Efficacy, Pharmacokinetics and Immunogenicity of BMS-188667 Administered to Subjects with Relapsing-Remitting Multiple Sclerosis	Terminated	Drug: Placebo Drug: BMS 188667 (Abatacept)	Phase 2
NCT01116427	A Phase II, Randomized, Double-blind, Parallel-group, Placebo-controlled, Multicenter Study to Evaluate the Safety and Efficacy of Abatacept in Adults with Relapsing-remitting Multiple Sclerosis	Completed	Biological: abatacept Drug: Placebo	Phase 2

## Data Availability

Not applicable.
